# Healthcare professionals’ experiences with the SARA robot in long-term care for people with dementia and people with intellectual disabilities

**DOI:** 10.1177/20552076251375530

**Published:** 2025-09-24

**Authors:** Nikée PA Zuurbier, Hanneke JA Smaling

**Affiliations:** 1 Public Health and Primary Care, 4501 Leiden University Medical Center, Leiden, Zuid-Holland, the Netherlands; 2 University Network for the Care Sector Zuid-Holland, Leiden University Medical Center, Leiden, Zuid-Holland, the Netherlands

**Keywords:** Robot, dementia, intellectual disability, healthcare professionals, long-term care

## Abstract

**Objectives:**

To explore the user experiences of healthcare professionals working with Social & Autonomous Robotic Health Assistant (SARA), a humanoid social robot, in long-term care (LTC) for people with dementia and people with intellectual disabilities (IDs). Secondarily, barriers to and facilitators for the implementation of SARA in LTC were identified. Lastly, the usability of the robot was explored.

**Design:**

A qualitative design using semi-structured interviews. The System Usability Scale (SUS) was used to explore the usability of the robot.

**Setting and participants:**

Seven healthcare professionals from 2 Dutch LTC organizations (one for dementia care and one for ID care) participated.

**Methods:**

Online semi-structured interviews were conducted. The SUS was completed online before the interview. Data were analyzed thematically using both an inductive and deductive approach.

**Results:**

Three themes were identified: 1) acceptance of SARA by all involved, 2) impact of SARA on all involved, and 3) user-friendliness. The acceptance of SARA among healthcare professionals grew over time. SARA may enhance job satisfaction and enjoyment. The reported impact on residents was predominantly positive. The few negative effects were seen when offered activities did not match the situation or the resident's needs. Although implementation required a time investment, healthcare professionals saw the potential of SARA to save time. Barriers to and facilitators for the implementation of SARA were identified: adequate education about working with SARA, high motivation among staff, and support from the supplier were reported as the most important facilitators. The usability was considered marginally acceptable. Participants provided recommendations to further improve SARA.

**Conclusions and implications:**

The user experiences with SARA were predominantly positive, making the robot a promising aid for healthcare professionals in delivering high-quality, personalized care in LTC. Quantitative studies are needed to assess the effectiveness of SARA. The usability of the robot may be improved by adhering to the recommendations when upgrading SARA.

## Introduction

The need for long-term care (LTC) increases with the growing aging population.^1-4^ The LTC is struggling with labor shortages, high staff turnover, and intense workload.^5^ However, adequate staffing and person-centered care are essential for maintaining high-quality care.^6-8^ To address these challenges, the United Nations recommends investing in LTC and new technologies.^1^ Care robots are a promising innovation to address these challenges in LTC.^9,10^ Robots may aid healthcare professionals, while also benefitting residents. Two growing groups within LTC that could benefit from social care robots are people with dementia and people with intellectual disabilities (IDs).^11-15^ Social robots can help with daily tasks, guide physical exercises, improve interaction, and provide emotional support.^9,16-18^

Several studies have found positive effects of social robots on nursing home residents with dementia.^19-21^ However, heterogeneous populations, intervention characteristics, and measured outcomes make it difficult to generalize the results.^21^ Also, research on adults with ID in LTC remains scarce. The few exploratory small-scale studies with adults with ID demonstrated robots may improve social engagement, support learning, increase physical activity, and reduce loneliness.^22-25^ These promising findings encourage further investigation on the use of social care robots in LTC.

So far, most studies examining the impact of robots in LTC have focused on residents. A study with the Zora robot in Dutch nursing homes found working with social care robots could improve work pleasure.^26^ However, another study found staff feared to be replaced by robots.^27^ These limited mixed findings highlight the need for further research on the impact of social robots on staff.

Despite potential benefits, implementing social robots in LTC remains challenging. Healthcare professionals have raised concerns about dehumanizing care, learning to operate robots, and difficulties faced by the target population.^20,28^ Another barrier for implementation is a complex innovation, underscoring the importance of prioritizing usability. Strategies to increase implementation success include careful prototyping for clinical integration, education, and staff engagement.^29,30^ Healthcare professionals play an essential role in implementation success, making it crucial to examine their experiences with working and implementing the robot.

The Social & Autonomous Robotic Health Assistant (SARA) is a social robot used in LTC for people with dementia and people with ID.^31^ The SARA robot was developed through a collaborative European innovation project, as an innovation activity of the European Institute of Innovation and Technology Digital.^32^ SARA can stimulate and calm residents by using personalized content, making it a promising intervention to support healthcare professionals in proving person-centered, high-quality care. This study explored healthcare professionals’ user experiences with SARA in LTC for people with dementia and people with ID. Facilitators for and barriers to SARA's implementation are investigated and SARA's usability is explored.

## Methods

### Design

This qualitative study used semi-structured interviews, allowing in-dept exploration of user experiences.^33,34^ SARA's usability was explored with a questionnaire.^35^ Data was collected between January and March 2024. COREQ guidelines were used to report results.^36^

### Recruitment

Participants were recruited via convenience sampling, as SARA was not widely used in LTC. One organization (dementia) was recruited via the researchers’ network, while the second (ID) was recruited via SARA's supplier. Researchers contacted the organizations via email using an information letter. Next, the organization's contact person invited healthcare professionals to participate using the information letter. Inclusion criteria were age ≥ 18, fluency in Dutch, at least 6 months of work experience with people with dementia or people with ID in intramural LTC, and experience with SARA or its implementation. Of the seven invited participants, one was unable to participate due to feeling unwell on the day of the interview. A colleague participated in her place.

### Setting

Dutch LTC-facilities provide 24-h support with activities of daily living (ADL) and specialized care from an on-site interprofessional team led by an elderly care physician (dementia-setting) or ID-physician, recognized medical specialties in the Netherlands.^37-39^ The majority of nursing home residents with dementia in the Netherlands typically present with moderate to severe stages of the condition.^40,41^ People with ID residing in LTC in the Netherlands primarily consists of individuals with moderate to severe IDs, often accompanied by challenging behavior. Most people with mild ID and people with mild dementia live in the community, those in institutional settings generally require intensive and continuous support.^40,42^

### SARA: Social & Autonomous Robotic Health Assistant

**Figure 1.**
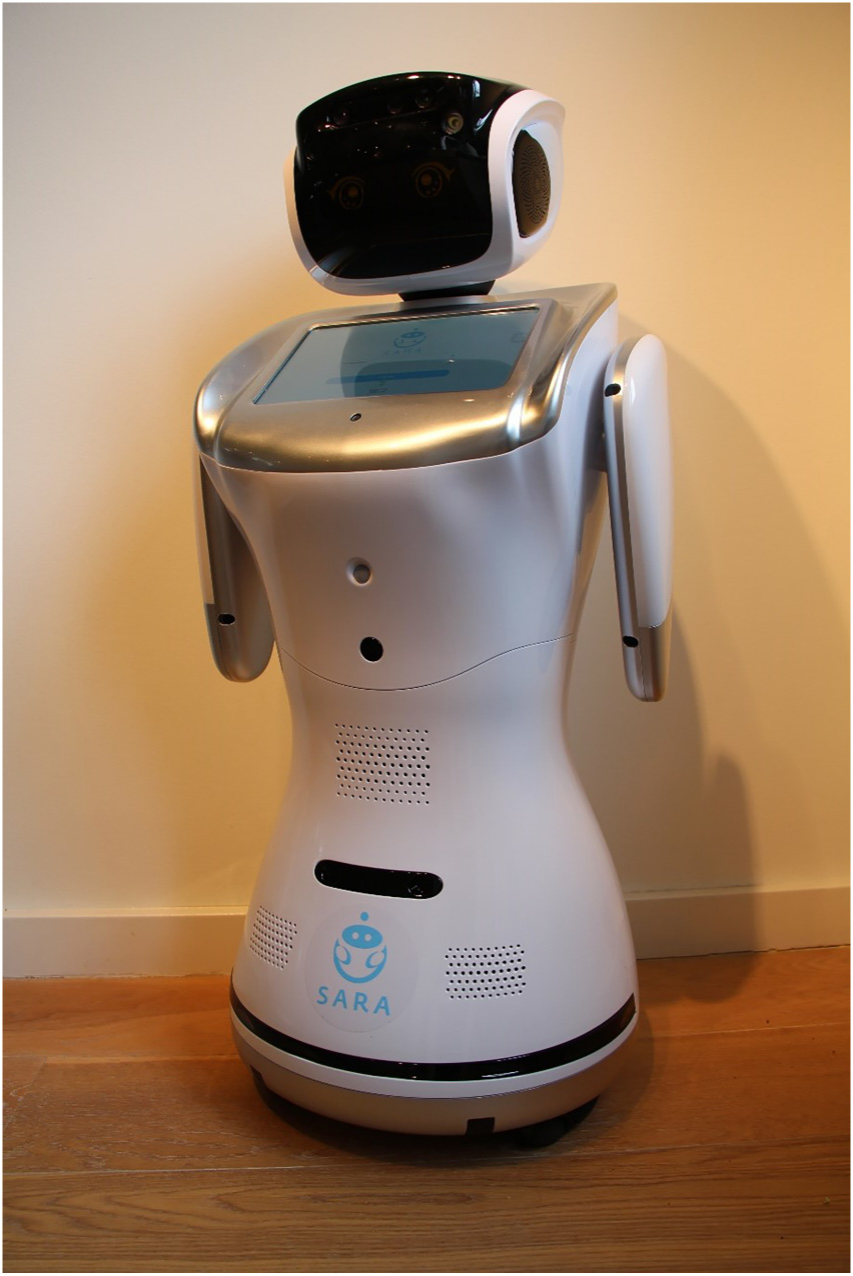
SARA one. SARA: Social & Autonomous Robotic Health Assistant.

**Figure 2.**
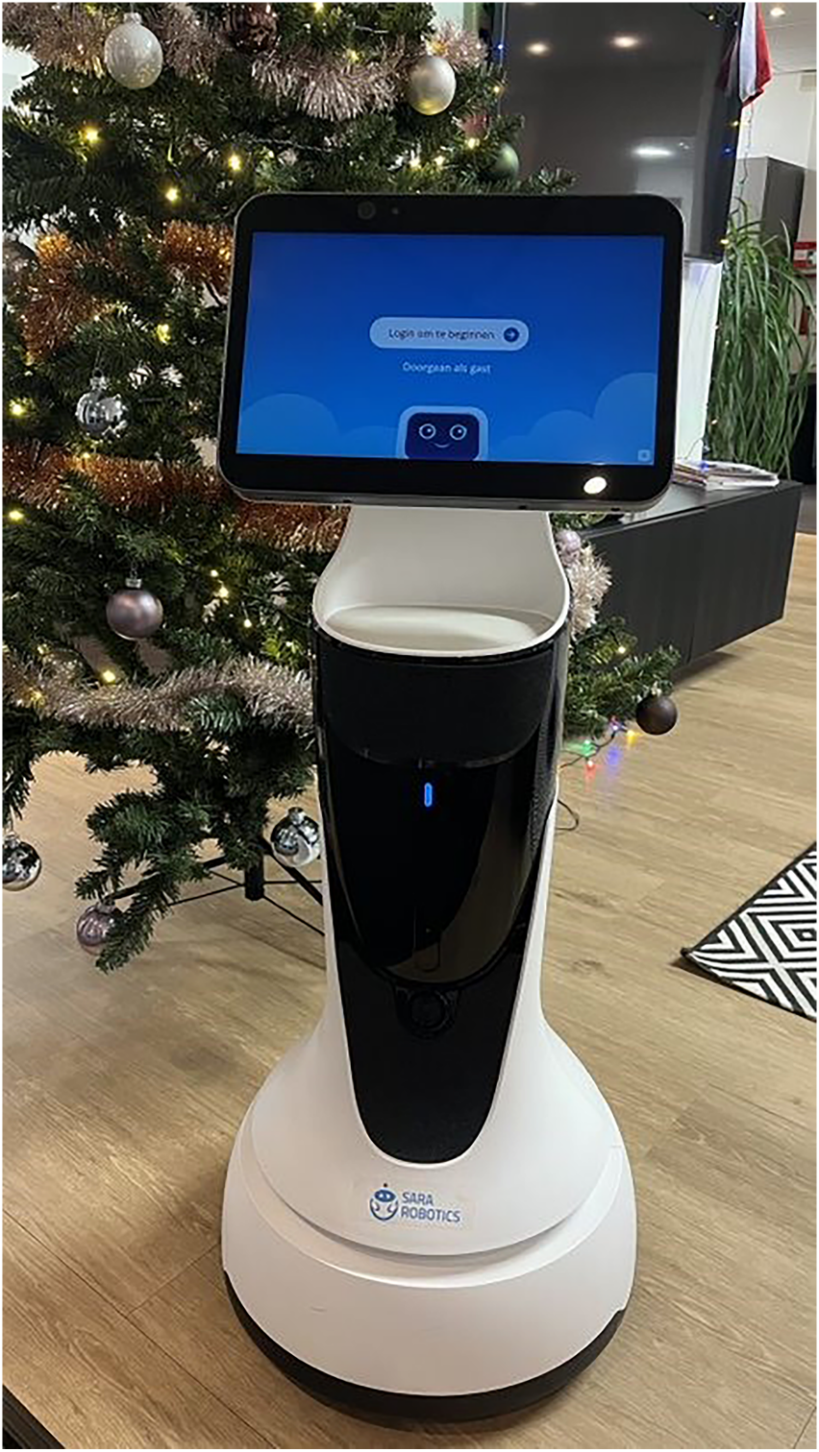
SARA star. SARA: Social & Autonomous Robotic Health Assistant.

The robot was integrated into routine care practices. Care teams were responsible for selecting residents and formulating individualized goals for the robot's use. Within the dementia care organization, the “SARA star” was employed, while the ID care organization utilized the “SARA one.”^31^ Both robots feature customizable programs. “SARA one” (see Figure 1) functioned as a prototype for the development of “SARA star” (Figure 2). User experiences with “SARA one” were evaluated by the supplier, and the feedback was incorporated into the design and functionality of the “SARA star.” Further technical specifications and details about the robots are provided in Supplement 1.

### Data collection

Before the interview, participants completed an online questionnaire via Castor Electronic Data Capture (Amsterdam, Netherlands), including informed consent, demographics, and the Dutch version of the System Usability Scale (D-SUS).^35^ The D-SUS measures the usability of systems using 10 items scored on a five-point-Likert scale. An overall score between 0 and 100 is computed, with <50 indicating unacceptable usability, 50–70 marginal usability, and ≥70 acceptable usability.^35,43,44^ SUS was developed in 1986 by Digital Equipment Co Ltd., Reading, United Kingdom.

Semi-structured interviews were conducted via Microsoft Teams by three female interviewers: HS (psychologist) supervising two psychology interns (SW, ELG). The interns were trained in interviewing techniques, observed an interview, and received feedback on their first interview. No others were present during the interview besides the interviewee and the researcher(s). No prior relationships existed between researchers and participants.

At the start of the interview, the interviewer introduced themselves by disclosing their name, occupation and role in the study. An interview guide with open-ended questions and prompts focused on user experiences and barriers to and facilitators for SARA's implementation was used (Supplement 2). Participants were also asked to rate their satisfaction with SARA on a scale of 1 to 10 during the interview. The interview guide was not pilot-tested. Interviews were recorded and transcribed verbatim. Interviewers made field notes during the interview, including their personal reflection on the interview. Participants received an interview summary for member-checking.^36^ No repeat interviews were conducted.

### Analysis

Two researchers (HS, NZ, MB; all female) coded each interview independently, followed by a consensus meeting. A third researcher mediated in case of disagreement. The initial code tree was developed inductively based on the first three transcripts, with additional codes added during the coding process if needed (Supplement 3).

To explore the user experiences, an inductive thematic analysis was chosen in order to provide a rich description of the gathered data.^45^ To analyze barriers and facilitators to implementation, a deductive thematic analysis was applied using the Consolidated Framework for Implementation Research (CFIR). This validated framework assesses contextual factors influencing implementation.^29,45,46^

NZ (MSc-medical student with experience with dementia care) performed the analyses following the 6 steps described by Braun & Clarke^45^ under supervision of HS. HS and NZ collaborated on theme development, and themes were presented to the research team for feedback. Coding and analyzing was supported by ATLAS.ti version 23.2.3.27778 (Scientific Software Development GmbH, Berlin, 2017). D-SUS data were analyzed using SPSS 29 (IBM, 2024).^47^

Typical data saturation for homogenous groups occurs within 6 to 12 interviews. Data saturation was defined as theoretical saturation—the point at which no new data emerged to develop category properties.^48^ No power analysis was performed for usability data, as the analysis was exploratory.

## Results

The seven interviews lasted between 28 to 54 min (median 32; IQR 28–37). Data saturation was reached as the last interview did not provide new codes to the categories. Table 1 contains participant characteristics.

**Table 1.** Participant characteristics.

ID	Age	Gender	Setting	Profession	Years of work experience in LTC^a^	Version of SARA^b^	SUS-score^c^	Satisfaction^d^
1101	46	Female	Dementia	Nursing assistant and innovation expert	15	SARA star	75	5
1102	23	Female	Dementia	Innovation expert	1.5	SARA star	67.5	6
1103	30	Female	Dementia	Nurse	10	SARA star	70	7
1104	36	Female	Dementia	Nursing assistant	18	SARA star	70	7.5
2201	56	Male	Intellectual disability	Director of operations	19	SARA one	50	8
2202	29	Female	Intellectual disability	Team manager	9	SARA one	82.5	8
2203	34	Female	Intellectual disability	Nursing assistant	8	SARA one	50	6

a LTC = long-term care.

b SARA = Social & Autonomous Robotic Health Assistant.

c SUS-score = System Usability Scale – score; possible range 0–100, with <50 unacceptable usability, 50–70 marginal usability, > 70 acceptable usability.

d Satisfaction score on a scale from 1 to 10.

### User experiences

**Figure 3.**
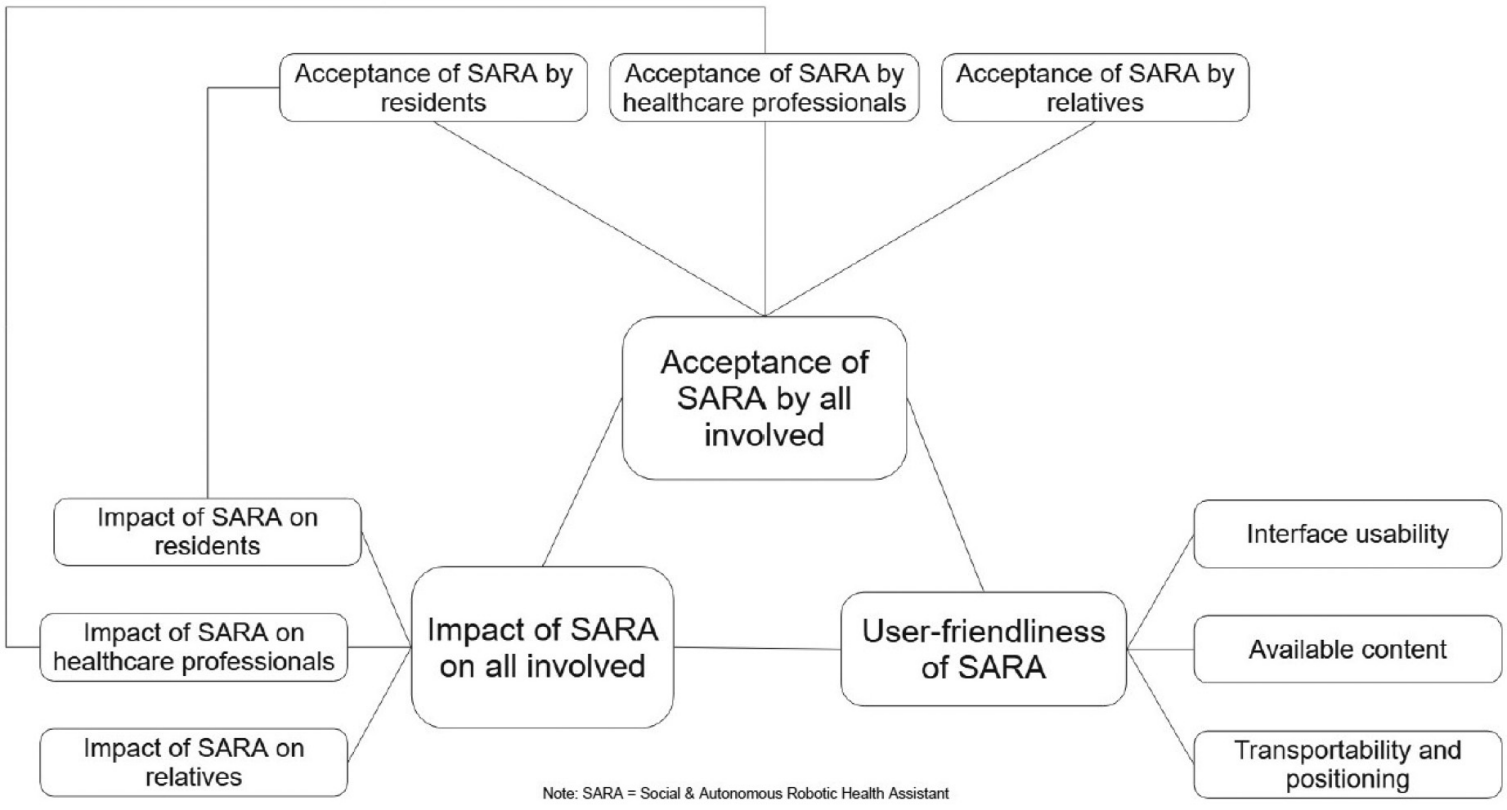
Interaction of themes and categories of the user experiences of healthcare professionals working with the SARA robot in long-term care.

Three themes were identified regarding user experiences: 1) acceptance of SARA by all involved, 2) perceived impact of SARA on all involved, and 3) user-friendliness. Table 2 provides an overview of the developed themes and the categories. Interrelations between themes are presented in Figure 3.

**Table 2.** Themes for user experiences working with SARA^a^ in long-term care.

Themes	Categories	Codes
Acceptance of SARA by all involved	Acceptance of SARA by healthcare professionals	Resistance of adoption by older or digitally illiterate healthcare professionals. A positive shift in acceptance by healthcare professionals over time.
	Acceptance of SARA by residents	No acceptance of SARA by resident. Fluctuating acceptance of SARA by resident, depending on the moment or the day. Immediate acceptance of SARA by resident.
	Acceptance of SARA by relatives	None of the relatives opposed SARA's introduction.
Impact of SARA on all involved	Impact on healthcare professionals	Mixed impact on workload and burden. Increased job satisfaction. Being able to now provide extra care, such as daily physiotherapy, which they usually lack time for.
	Impact on residents	Calming residents when agitated or distressed. Exacerbation of distress due to overstimulation by SARA. Distracting residents during ADL^b^-care. Stimulating physical activity. Refusal of residents to engage in activating programs. Providing entertainment. Stimulating social engagement among residents. Enhancement of independency.
	Impact on relatives	No effect as relatives did not interact with SARA. Potential effect if interaction with SARA could be a communal activity with their relative. Potential positive impact on relatives’ perception of healthcare professionals.
User-friendliness of SARA	Interface usability	Easy to use for the healthcare professionals. Challenging to read white text Hard to use the touchscreen for residents.
	Available content	Wide range of available content. Content does not always match with cognitive abilities of resident. Programming personalized content takes too much time.
	Transportability and positioning	Mixed opinions on current ease to move the robot. Height is non-adjustable, making SARA less suitable for bedridden residents.

a SARA = Social & Autonomous Robotic Health Assistant.

b ADL = activities of daily living.

#### Acceptance of Social & Autonomous Robotic Health Assistant by all involved

##### Acceptance by healthcare professionals

Staff's acceptance of SARA in the dementia and ID group varied.

‘Take digital illiterates … they tend to avoid SARA, even though it is not actually difficult to use.’ [ID, 2203]

One interviewee from the ID-setting highlighted apprehension among colleagues caused by fear of being replaced by SARA. Others described a positive shift in their opinion of SARA over time caused by coworkers’ enthusiasm and satisfaction with the supplier's support during problems.

##### Acceptance by residents

The reported effects of SARA on residents depended on the resident and situational factors. Some residents rejected SARA. Others, especially those with dementia, showed a mixed interest in the robot. However, many residents readily accepted SARA. One interviewee was concerned that some residents with ID might become overly attached to SARA, potentially leading to diminished responsiveness to healthcare professionals.

##### Acceptance by relatives

Prior to implementation, relatives were informed about SARA. Interviewees mentioned family caregivers expressed no explicit objections to its introduction.

#### Impact of Social & Autonomous Robotic Health Assistant on all involved

##### Impact on healthcare professionals

Healthcare professionals in both settings needed time to integrate the robot into their workflow. Some interviewees reported a saving of time and energy after getting used to working with SARA, while all interviewees reported potential time savings in the future:

‘SARA helped by taking over a portion of care responsibilities from staff. SARA has been supportive in that regard, serving as an aid, much like an electric bicycle, which requires less effort to pedal.’ [ID, 2202]

Interviewees reported SARA improved job satisfaction. Reasons included offering residents something “extra” and seeing the joy SARA can bring them. Furthermore, SARA provided a way to offer personalized care healthcare professionals lack time for:

‘The physiotherapists ask us to practice certain movements daily with residents. In practice, that is just not feasible. SARA can take over that task by showing the physiotherapist's video that we had uploaded.’ [ID, 2202]

##### Impact on residents

Interviewees reported various effects of SARA on residents, depending on the program used and the residents’ needs. In both settings, interviewees mentioned the calming influence of the robot's music and videos on residents experiencing agitation or distress:

‘She was restless all afternoon. We then introduced SARA. Since she used to be a farmer, we played videos of nature, horses, and cows. You could gradually see her becoming calmer.’ [Dementia, 1104]

Sometimes SARA had no effect or exacerbated distress. This adverse reaction was only reported in the dementia group and was mostly attributed to additional sensory stimulation introduced by SARA. Moreover, calming effects were less when the resident walked around when agitated, since SARA was stationary.

In the dementia-setting, music and video programs were utilized to distract residents during ADL, thereby minimizing resistance to care:

‘We had a resident who would complain a lot during morning care. We created a program for her with personalized songs, and she sang along. In the meantime, we could carry out the entire morning care routine, and she was genuinely engaged.’ [Dementia, 1101]

Stimulating effects on physical activity using programs or videos recorded by the physiotherapist were reported in both groups. In the ID setting, challenges were reported. One resident experienced stress because they felt obligated to engage with SARA, while another outright refused to respond to the robot:

‘We also used movement exercises with another resident, but they are intellectually at a higher level, and they literally had the attitude of, ‘Yeah, what's this thing doing here? If you want to talk to me or want me to exercise, then you should come yourself. You must work for your money.’ So, not everyone will accept SARA, and we accept that too.’ [ID, 2202]

In both settings, SARA entertained residents with music, videos, quizzes, and cognitively engaging games. In the dementia group, quizzes facilitated group activities, promoting social engagement. Entertainment programs had no observed effect when content did not align with the residents’ needs and interests.

Interviewees reported SARA improved residents’ independence by enabling them to engage in activities autonomously, reducing reliance on healthcare professionals. In some cases, perceived effects of interacting with SARA manifested within minutes. In others, after several weeks.

##### Impact on relatives

In the ID group, interviewees did not see an impact on relatives, as relatives did not interact with SARA. However, within the dementia group, a potential effect was identified, suggesting introducing SARA to family and allowing them to interact with the robot alongside their relatives could serve as a communal activity. Additionally, a potential positive impact was noted in how relatives perceive healthcare professionals:

‘We have sometimes asked if family would like to record personalized content, such as a video or tell a story [to upload on SARA]. And yes, that does have an impact on them, but I think it's mostly in a positive way because they see that we are genuinely exploring other ways to provide attention and comfort to their loved ones.’ [Dementia, 1102]

#### User-friendliness

##### Interface usability

All interviewees found SARA's interface easy to use. They highlighted the well-functioning touchscreen, user-friendly buttons, and simplicity of initiating programs. Some residents with dementia had trouble reading the text on the interface when presented in white font. In the ID setting, some residents could operate SARA independently; however, these residents faced challenges with the touchscreen interface:

‘You must tap in a specific way; otherwise, it does not register. This can be very frustrating for residents.’ [ID, 2202]

##### Available content

Opinions on SARA's available content varied. Positive aspects included the ability to select content from YouTube (SARA one), access a range of quizzes, upload personalized videos, and to customize programs. However, interviewees complained about the time required to program personalized content and limited variety of available material. Additionally, content did not always align with residents’ cognitive abilities.

##### Transportability and positioning

Interviewees expressed mixed opinions regarding the ease to move SARA. Currently, SARA can be moved manually, which was generally perceived as favorable. However, some participants in the dementia setting believed the robot should operate autonomously and be controlled remotely. Others in the ID setting argued such autonomy might be unpredictable for residents and therefore undesirable. A reported limitation was the inability to adjust SARA's height, meaning people who are bedridden may not be able to see SARA.

No notable differences in the user-friendliness were reported by healthcare professionals who interacted with either the SARA “one” or the “star” version.

#### Facilitators for and barriers to implementation

An overview of factors impacting implementation of SARA categorized using the domains of the CFIR was presented in Table 3. Education, motivation, and supplier support were key facilitators for implementation. Some barriers were setting-specific: in the ID setting, these included insufficient education, job replacement fears, and poor Wi-Fi, while in the dementia setting decreased motivation due to SARA's slow innovation was reported. Healthcare professionals mentioned recommendations to deal with challenges encountered when working with and implementing SARA, see Table 4.

**Table 3.** Facilitators for and barriers to the implementation of SARA^a^ in long-term care.

Themes from CFIR^b^	Facilitators	Barriers
Innovation [*The subject of implementation*]	A strong partnership with the supplier.^e^ Feedback regarding improvements to the robot is acknowledged and actively addressed by the supplier . ^e^	Slow innovation of robot leading to a decrease in motivation among healthcare professionals . ^c^ Malfunctioning of robot leading to a decrease in motivation among healthcare professionals . ^e^
Outer setting [*The broader environment encompassing the Inner Setting*]	Permission from relatives to use the robot with residents.^e^	Holiday season resulting in a relatively small number of regular staff . ^d^
Inner setting [*The specific environment where the innovation is introduced*]	Strong Wi-Fi connection.^d^ Ample education about how to use the robot for staff to be able to work with the robot efficiently.^e^ Motivation and support of management . ^e^	Weak Wi-Fi.^d^ No education about the robot provided to staff by supplier or organization.^d^ Lack of time for the implementation . ^d^
Individuals [*The roles and attributes of the people involved*]	Curiosity of residents to interact with the robot . ^c^ Curiosity of healthcare professionals to work with the robot . ^e^ High motivation amongst healthcare professionals to implement the robot . ^e^ A sense of pride amongst healthcare professionals to attribute to the innovation of the robot . ^e^ Healthcare professionals seeing a potential in the innovation to be valuable in the future . ^e^	Fear of job replacement by robots amongst healthcare professionals.^d^ Low motivation amongst individual healthcare professionals . ^e^ A lack of acceptance of the robot by residents.^e^
Implementation process [*The actions and strategies employed to introduce the innovation*]	Starting with a small pilot that can be extended when successful . ^d^ Ample preparation by the organization before implementation . ^d^ A structured implementation plan provided by the healthcare organization to the healthcare professionals . ^e^ Innovation teams to guide implementation.^e^ Reflection and evaluation moments amongst healthcare professionals to guide implementation . ^e^	Vague or unclear plan of implementation.^d^ Vague or unclear communication about the plan of implementation.^d^

a SARA = Social & Autonomous Robotic Health Assistant.

b Themes from: Updated Consolidated Framework for Implementation Research domain and construct definitions.^29^

c reported by participant from dementia setting.

d reported by participant from intellectual disabilities setting.

e reported by participants from both settings.

**Table 4.** Recommendations by the interviewees for the challenges identified when working with SARA in long-term care with residents with dementia and residents with intellectual disabilities.

Challenge	Recommendation
Healthcare professionals need to get used to working with SARA^b^	Provide adequate education about using SARA during implementation.^b^ Provide a clear plan for implementation.^b^
Residents can get overstimulated by SARA^a^	Make sure the volume between different videos or different types of content stays the same (overstimulation caused by loud noise).^a^
SARA can be hard to use for residents^c^	Make SARA react to movement e.g., waving.^a^ Make SARA voice-activated . ^b^ Provide a special pen for the touchscreen . ^b^ Make the touchscreen more sensitive . ^c^ Use different fonts to make the letters easier to read for residents . ^a^
A discrepancy between the content provided and the needs of the user^c^	Expand the selection of games and quizzes available on SARA.^c^ Offer various difficulty levels to match with the level of cognitive impairment.^c^ Improve the personalized programs to better meet the needs and wants of residents.^c^
Programming the personalized profiles takes a lot of time^b^	Streamline the procedure by utilizing templates based on similar cases.^b^
Deploying SARA requires a significant amount of time^c^	Implement the option for remotely controlling SARA^a^ Make SARA autonomous.^a^ Make SARA able to recognize emotions and to react appropriately to those emotions . ^a^
Other recommendations	Deploy SARA 24-h per day to make the robot more profitable.^b^ Approximately 1 robot per 10 residents is advised.^c^

a reported by participant from dementia setting.

b reported by participant from intellectual disabilities setting.

c reported by participants from both settings.

#### Usability and satisfaction

On average, participants rated their satisfaction with SARA for LTC with 6.8 (median 7, IQR 6–8). Usability-scores are presented in Table 1. The mean SUS-score was 66.4 (median 70, IQR 50–75), indicating a marginal overall usability level. None of the participants deemed SARA's usability unacceptable.

## Discussion

This study examined user experiences, facilitators of and barriers to implementation, and usability of SARA in LTC for people with dementia and people with ID. Three themes were identified: 1) acceptance of SARA by all involved, 2) impact of SARA on all involved, and 3) user-friendliness. Most healthcare professionals had positive experiences working with SARA and mentioned SARA potentially saving them time with care tasks. The match between residents’ needs and interests, and offered content on SARA was essential for a positive impact. All mentioned barriers to and facilitators of the implementation fitted in the CFIR domains innovation, outer setting, inner setting, individuals, and implementation process. SARA's usability was (marginal) acceptable.

This study found healthcare professionals’ acceptance of SARA increased over time due to coworker's enthusiasm and effective supplier support. Factors contributing to increased acceptance align with the CFIR.^29^ Prior to implementation, concerns regarding job replacement were reported, which is consistent with existing literature.^27^ However, following direct experience with SARA, these concerns were no longer expressed. This suggests that fear of job replacement may be more strongly linked to uncertainty and unfamiliarity than to actual interactions with social robots. Providing staff with opportunities to engage with SARA, along with explicitly addressing concerns about role displacement during the introduction phase, appears to be critical for successful implementation.

Working with SARA can enhance job satisfaction and increase workplace enjoyment, which aligns with previous research on care robots.^26^ This finding is especially relevant in light of growing concerns about job satisfaction in LTC. SARA's impact on perceived workload was mixed. During initial implementation, SARA required a time investment. Later, SARA demonstrated potential to improve efficiency and ultimately save time. All interviewees recognized its future time-saving potential, which is particularly valuable given ongoing labor shortages and high work-burden.^5^ Integration of SARA into daily practice allowed healthcare professionals to optimize their workflow and enabled them to provide additional attention to residents facilitating person-centered care.

A calming effect of SARA on residents with ID was noted, which is particularly significant, since 37–86% of individuals with ID exhibit challenging behavior. Such behavior negatively impacts other residents, complicates caregiving, and contributes to increased sick leave and staff turnover.^49^ A recent study suggests that SARA has the potential to reduce both challenging behaviors in residents with dementia and the associated burden for staff, when the robot's use is tailored to align with residents’ individual preferences, needs, and contextual factors.^50^ Future research should explore whether SARA can reduce challenging behavior, absenteeism, and staff turnover.

Our findings indicate SARA can enhance residents’ independence in daily activities, particularly among individuals with ID and those with dementia who have relatively preserved cognitive functioning. SARA grants them access to meaningful activities, thereby potentially improving quality of life. Moreover, SARA may also foster self-determination, which is directly linked to quality of life in people with ID living in LTC.^6,7,51,52^ Increased resident independence may alleviate healthcare professionals’ workload. More research is needed to examine SARA's long-term impact on residents’ quality of life and time healthcare professionals spend on care tasks. A potential next step in upgrading SARA could involve incorporating step-by-step guidance for daily tasks to further promote independence and autonomy.

For a positive impact on residents, it was essential that SARA's program and content were tailored to the individual. A mismatch between the resident's needs and SARA's content resulted in no or adverse effects. Improving SARA's ability to facilitate personalized activities and content aligns with LTC recommendations for person-centered care, promoting well-being in people with dementia.^53^

SARA could have a calming effect, stimulate physical activity, or distract residents based on the chosen content, which was in line with other studies in LTC for people with dementia.^9,16-18^ Moreover, enhanced communication among residents with dementia was reported similar to other studies.^16,20,25^ This outcome is particularly significant, as maintaining social contact is frequently cited as a key factor in enhancing quality of life in LTC settings.^6,7^ Furthermore, a recurrent mentioned argument against using robots in healthcare is the loss of social engagement.^20,28^ However, our findings suggest social robots may enhance social engagement when used as a group activity.

All identified facilitators and barriers to SARA's implementation aligned with the existing framework and previous research on the implementation of social robots in LTC.^29,30,46^ Important factors for implementation were education, Wi-Fi-connection, motivation, implementation plan, time to prepare for the implementation, and the functioning of the robot (including supplier support). Acceptance among older and digitally illiterate healthcare professionals was harder to achieve. Future research should explore the needs and support required during implementation for these specific groups to enhance adoption and implementation success.

### Strengths and limitations

Strengths of this study include using in-depth semi-structured interviews allowing flexible and nuanced exploration of participants’ experiences, and including two settings contributing to the richness of data. The diverse group of healthcare professionals allowed experiences from different viewpoints within the organizations to be gathered. Recommendations for improving SARA were gathered in a clinical setting, ensuring enhancements are grounded in real-world clinical practice. This approach assures future improvements to be tailored to the work environment in which they are intended to function, which will help with implementation.^29^

A limitation is the inclusion of a single organization per setting, all of which were already working with SARA, restricting the generalizability of the findings. Researchers were not involved in the implementation process. To enhance the robustness and external validity of the results, future research should consider a larger sample including multiple organizations, potentially across different countries. Also, family caregivers were not actively involved in implementing SARA. Although potential positive effects on family were mentioned, future research should explore the impact of SARA on relatives. SARA's acceptance by family caregivers must be examined, as limitations in decision-making capacity in residents in LTC make proxy/family consent essential.

## Conclusions and implications

This study was the first study exploring healthcare professionals’ user experiences with SARA in LTC with residents with dementia and residents with ID. To fully benefit from SARA's potential positive impact on residents, a person-centered approach is essential taking into account the situation and needs and preferences of the resident. After learning how to work with SARA, it increased job satisfaction and could potentially save healthcare professionals time. Successful implementation in LTC requires adequate education on SARA's operation, its role as an aid rather than a staff replacement, and a clear implementation plan. Although SARA's usability was (marginal) acceptable for residents with dementia and residents with ID in LTC, usability can be further improved by reducing the time needed to program personalize content, including more content for people with varying degrees of cognitive abilities, and making SARA easier to use for residents. Although there is room for improvement, SARA may be considered a promising aid to support healthcare professionals in delivering high-quality person-centered care.

## Supplemental Material

sj-docx-1-dhj-10.1177_20552076251375530 - Supplemental material for Healthcare professionals’ experiences with the SARA robot in long-term care for people with dementia and people with intellectual disabilities Supplemental material, sj-docx-1-dhj-10.1177_20552076251375530 for Healthcare professionals’ experiences with the SARA robot in long-term care for people with dementia and people with intellectual disabilities by Nikée PA Zuurbier and Hanneke JA Smaling in DIGITAL HEALTH

sj-pdf-2-dhj-10.1177_20552076251375530 - Supplemental material for Healthcare professionals’ experiences with the SARA robot in long-term care for people with dementia and people with intellectual disabilities Supplemental material, sj-pdf-2-dhj-10.1177_20552076251375530 for Healthcare professionals’ experiences with the SARA robot in long-term care for people with dementia and people with intellectual disabilities by Nikée PA Zuurbier and Hanneke JA Smaling in DIGITAL HEALTH
