# Supplementary material for: Healthcare professionals’ experiences with the SARA robot in long-term care for people with dementia and people with intellectual disabilities
Source: Digit Health. 2025 Sep 24;11:20552076251375530. doi: 10.1177/20552076251375530 (PMC12461080; doi:10.1177/20552076251375530)
Supplement: sj-docx-1-dhj-10.1177_20552076251375530 - Supplemental material for Healthcare professionals’ experiences with the SARA robot in long-term care for people with dementia and people with intellectual disabilities [file sj-docx-1-dhj-10.1177_20552076251375530.docx]

**Supplementary Materials**

Healthcare professionals’ experiences with the SARA robot in long-term care for people with dementia and people with intellectual disabilities.

Nikée P.A. Zuurbier & Hanneke J.A. Smaling

**Supplement 1. Description of the robots**

**SARA one** includes the Sanbot Elf hardware (Sanbot Innovation Technology, 2024) using the SARA 2.0 software. It has a 10.1-inch display. The robot has a size of 90 cm x 42 cm x 33 cm and weighs 19 kilograms. This SARA is a humanoid robot that can move its arms and head with eyes that can represent emotions. The arms can also differ in color depending on the preferences of the user. It can recognize the resident based on face recognition through the 3D camera and accordingly play content (e.g., audio stories, (gymnastic) videos, quizzes, or music) that corresponds to the right resident profile and their goals. The face recognition was not enabled in this study.

**SARA star** uses the hardware of the Mini bot developed by Orionstar Europe (OrionStar Europe, 2024). The robot uses the 3.0 version of the SARA software (SARA Robotics, Eindhoven). Its screen is fourteen inches and has a resolution of 1920px*1080px. The robot has a height of 100 cm and a width of 41 cm, weighs 21 kilograms, and operates on a stable Wi-Fi or 4G/5G-connection. There are several sensors embedded in the hardware, such as a 6-mic circular array, HD wide angle dual camera, lidar navigation, and a depth camera. However, these were not enabled in this study.

**Differences in software**

The content of the software version 3.0 has been developed by the supplier SARA Robotics (In Dutch: Zorgen met SARA). Several key differences between the software versions pertain to interaction design and personalization. Users of version 2.0 highlighted the importance of maintaining engagement through mid-session interaction. In response, version 3.0 introduces integrated mid-session check-ins, implemented via supportive spoken prompts, personalized vocatives, and expressive facial cues. Additionally, the structure of individual programs has been enhanced. While version 2.0 required manual composition of sessions, version 3.0 supports the automatic generation of personalized sessions. This is achieved through the use of labeled content in conjunction with resident profiles that reflect individual preferences. The calendar functionality has also been expanded. An addition in version 3.0 is the “Zelf Doen” (“Do It Yourself”) feature, which allows for the creation of step-by-step guides tailored to specific activities. These guides are designed to support residents in independently performing structured tasks, thereby fostering autonomy and self-efficacy. Finally, version 3.0 offers enhanced system stability and improved performance in offline environments, reducing reliance on a continuous internet connection. The user interface has also been refined to provide a more intuitive and accessible experience. YouTube was only used in the SARA one (prototype). The new version uses fully licensed and/or in house developed content that is not based on YouTube.

**Supplement 2. Interview guide**

[highlighted in grey] = possible topics to further explore / probes

Could you please briefly introduce yourself?

[Profession, setting, location/unit]

Could you describe how the robot SARA was implemented at your unit/ward?

What went well during the implementation process?

What could have been improved during the implementation process?

[Education, satisfaction with process, room to experiment and reflect]

Were you involved in the decision-making process for implementing the SARA care robot, or do you know how that decision was made? [Decision-makers, decision-making process, down-top or bottom-up, considerations]

Were there any discussions about possible ethical issues that might arise from using a robot with the target group?

[Decision-making capacity vs. coercion, data use, storage and access, dehumanizing care]

Do you remember what your expectations were regarding the SARA robot?

Could you tell us something about how you use the SARA robot?

[With whom, frequency, how many robots per unit, individual vs. group, decision-making resident selection]

What are you the minimal requirements you need to start using SARA?

For what purposes is SARA used? [Activation, calming, combating loneliness, engagement]

On average, how often do you work with SARA?

[Changes in usage over time, reason for the change]

If they no longer work with SARA, ask why and when they stopped using it. [reason to restart use SARA, minimal requirements to start working with SARA]

When is the robot usually used? [time of day; during which care tasks]

In general, how often per day is the robot used?

Are there also times when SARA is deliberately not used? [Why?]

Based on your initial experiences with the SARA robot, how many robots would be desirable in your unit for providing optimal care?

*The following questions are about the use of the SARA robot. The interviewer first lets the interviewee describe the situation and then asks probing questions.* [Think about: how often it occurs, interaction with the robot, functions/settings of the robot, positive and negative responses, impact on care]

Could you describe a situation where you successfully used the SARA robot in the care for a resident? [context – mechanism – result; what made it successful and why]

Could you describe a situation where you did NOT successfully use the robot in the care for a resident? [context – mechanism – result; why not successful]

Does the use of the care robot SARA have an impact on:

[residents; mood, behavior, cognition, social contact, interactions, self-reliance; staff, yourself; workload, job satisfaction, work pleasure, interaction with colleagues and family]

Does the robot affect how much time you spend on certain tasks?

[Time spent on mitigating challenging behavior, ADL, personal attention, etc.].

For a follow-up study, based on your experiences, how much time would be needed to detect a difference in the residents’ behavior based on the deployment of SARA?

What do you think of the user-friendliness of the robot?

What could be improved? What works well?

Is there anything that could have helped you to make learning to use the robot easier?

What is your opinion about the available features of the robot SARA?

[satisfied with, room for improvement]

What do you think of the robot’s controls?

[number of buttons, screen size, volume adjustments, etc.]

What is your opinion about the residents’ interaction with the robot?

[whether the resident was allowed to control the robot themselves, improvements]

How satisfied are you with SARA for the long-term care of the target group; [dementia or intellectual disabilities]? (on a scale of 1 to 10)

[Explanation for rating, added value of SARA for long-term care]

Do you have any other positive or negative experiences with the use of SARA that we have not discussed yet? [Decisive reasons for (not) using the SARA robot].

Is there anything else you would like to add to this conversation about your experience with the SARA robot?

**Supplement 3. Coding book**

**Implementation**

- Reason(s) for implementation [*start / continuation*]
  - Innovation SARA robot [*factors related to SARA; added value, collaboration with supplier, adaptability, expectations*]
  - Care gap [*staff shortage, increasing complexity, family involvement, aids for staff*]
  - Added value for residents
  - Improve quality
  - Cost reduction [*increase efficiency, business case*]
- Process of implementation [*used strategies and activities*]
- Decision-making process [*decision-makers, top-down vs. bottom-up, considerations*]
  - Preparing for the future
  - Liability in case of incident with robot [*insurance*]
- Deployment of robot
  - Target population [*group vs. 1-on-1; dementia, intellectual disabilities, change in population*]
  - Frequency [*per resident, day; duration; ratio staff: robots, etc*.]
  - Goals use [*why, when, what programs, what (not) take over from staff*]
  - Ethical considerations [*warm vs. cold care; care gap*]
- Requirements and barriers to and facilitators for implementation
  - Motivational factors [*enthusiasm, expectations*]
  - Organizational factors [*infrastructure, such as Wi-Fi, budget, staff shortage*]
  - Education [*information, training, learning from each other, joint evaluation*]
  - Support from management
  - SARA-related [collaborator with supplier, functioning of robot)
  - Consent family caregivers and residents [*acceptance / consult family*]
  - Added value for residents

**Experiences** [*Impact SARA on care; added value, perceived effects*]

- Impact on care-triangle
  - Impact on residents [*behavior, emotion, agitation, incl. duration for effect*]
  - Impact on staff
  - Impact on family caregivers
- User experiences
  - Factors related to the SARA robot [*features, limitations, soft-/hardware*]
  - Satisfaction [*rating on scale 1 to 10, explanation, potential, added value*]
  - Recommendations
  - Acceptance of SARA by stakeholders [*including objections for use*]

**Context** [*work experience, setting, when worked with SARA*]

**Other** [e.g., *sharing knowledge about robot in long-term care*]
